# Phosphorylation of eIF2α on Threonine 169 is not required for *Trypanosoma brucei* cell cycle arrest during differentiation

**DOI:** 10.1016/j.molbiopara.2016.03.004

**Published:** 2016

**Authors:** Carla Cristi D.C. Avila, Lori Peacock, Fabricio Castro Machado, Wendy Gibson, Sergio Schenkman, Mark Carrington, Beatriz A. Castilho

**Affiliations:** a Department of Microbiology, Immunology and Parasitology, Escola Paulista de Medicina, Universidade Federal de São Paulo, São Paulo, SP, Brazil; b Department of Clinical Veterinary Science, University of Bristol, Langford, Bristol BS40 5DU, UK; c School of Biological Sciences, University of Bristol, Bristol BS8 1TQ, UK; d Department of Biochemistry, University of Cambridge, Tennis Court Road, Cambridge CB2 1QW, UK

**Keywords:** RNAPII, RNA polymerase II, eIF2α, alpha subunit of eukaryotic initiation factor 2, *T. brucei*, Cell cycle arrest, Differentiation, Translation initiation, eIF2

## Abstract

The trypanosome life cycle consists of a series of developmental forms each adapted to an environment in the relevant insect and/or mammalian host. The differentiation process from the mammalian bloodstream form to the insect-midgut procyclic form in *Trypanosoma brucei* occurs in two steps *in vivo*. First proliferating ‘slender' bloodstream forms differentiate to non-dividing ‘stumpy' forms arrested in G1. Second, in response to environmental cues, stumpy bloodstream forms re-enter the cell cycle and start to proliferate as procyclic forms after a lag during which both cell morphology and gene expression are modified. Nearly all arrested cells have lower rates of protein synthesis when compared to the proliferating equivalent. In eukaryotes, one mechanism used to regulate the overall rate of protein synthesis involves phosphorylation of the alpha subunit of initiation factor eIF2 (eIF2α). The effect of eIF2α phosphorylation is to prevent the action of eIF2B, the guanine nucleotide exchange factor that activates eIF2 for the next rounds of initiation. To investigate the role of the phosphorylation of eIF2α in the life cycle of *T. brucei*, a cell line was made with a single eIF2α gene that contained the phosphorylation site, threonine 169, mutated to alanine. These cells were capable of differentiating from proliferating bloodstream form cells into arrested stumpy forms in mice and into procyclic forms *in vitro* and in tsetse flies. These results indicate that translation attenuation mediated by the phosphorylation of eIF2α on threonine 169 is not necessary for the cell cycle arrest associated with these differentiation processes.

## Highlights

• Pleomorphic *T. brucei* expressing an eIF2α phosphorylation site mutant were made.
• The mutation did not prevent normal arrest and differentiation into stumpy forms.
• Mutants differentiate into procyclic forms *in vitro* and in tsetse flies.

## 1 Introduction

Kinetoplastids are protozoa and a large number of species have evolved to infect humans and/or animals. Many of the pathogenic species have complex life cycles and have evolved to proliferate in different niches within one or more host through evolution of a series of developmental forms each adapted to an environment in the relevant invertebrate and, sometimes, vertebrate host. The differences between one developmental form and another include alterations in gene expression and cellular morphology. The transition from one developmental form to another has been used to investigate the regulation of these processes [1]. The best-characterised transition is from the proliferative mammalian bloodstream form to the insect midgut procyclic form in *Trypanosoma brucei*. The process includes two differentiation steps *in vivo*, first proliferating ‘slender' bloodstream forms differentiate to non-dividing ‘stumpy' forms arrested in G1 [2]. Second, in response to ingestion by a tsetse fly or environmental cues that mimic this event in culture, the cell cycle arrest is ended after a lag of 8–12 h and the trypanosomes re-enter the cell cycle and start to proliferate as procyclic forms [3]. Both steps include alterations in cell morphology and gene expression [4]. The stumpy form retains the ability to differentiate for at least two days and thus the cell cycle arrest can persist from the production of a stumpy form to several hours after the signal to differentiate [5]. Proliferating cells nearly always have higher rates of total protein synthesis than the arrested equivalent, and so the differentiation process is accompanied by a reduction in the overall rate of protein synthesis as the slender to stumpy bloodstream form differentiation occurs, followed by an increase as the cells proliferate as procyclic forms [6]. How is the overall rate of protein synthesis regulated in trypanosomes? The answer is not known for the differentiation associated arrest described above but has been investigated for procyclic cells that arrest due to heat shock [7]. On induction of heat shock, there is a rapid inhibition of initiation, but not elongation, of transcription by RNA polymerase II (RNAPII) and the steady state levels of most mRNAs decrease rapidly, probably via both reduced transcription and a general increase in mRNA turnover. However, cells in heat shock only retain viability for two to six hours; this is significantly shorter than the two or three days that stumpy cells retain viability [8], [9].

Selective transcriptional control by RNAPII is unlikely to play a role in the G1 arrest that occurs on differentiation to stumpy forms. In trypanosomes, genes occur in long tandem arrays, usually encoding functionally unrelated proteins, and are transcribed polycistronically from occasional transcription start sites; co-transcriptional processing results in individual monocistronic mRNAs. This lack of transcriptional regulation of individual genes means that the relative levels of individual mRNAs are determined post-transcriptionally. RNAPII transcription must continue in arrested stumpy cells as some mRNAs have increased levels [10]. The overall rate of protein synthesis has been measured in stumpy forms and it is reduced several fold reflected by a reduction in the number of polysomes suggesting that the rate of translation initiation is regulated [11].

In eukaryotes, one mechanism used to regulate the overall rate of protein synthesis involves the phosphorylation of serine 51 (S51) in the eIF2α. eIF2 is a trimeric G-protein that forms a ternary complex with the initiator methionyl tRNA that delivers the Met-tRNA to the pre-initiation complex. At the start codon, release of eIF2 occurs after GTP hydrolysis. The effect of eIF2α phosphorylation is to prevent the action of eIF2B, the guanine nucleotide exchange factor that activates eIF2 for the next rounds of initiation. This is turn reduces the amount of available ternary complex and the rate of translation initiation. The kinases that phophorylate eIF2α contain a catalytic domain attached to different regulatory domains. The best conserved are GCN2, which is activated by amino acid deficiency, and PERK which is activated by an increase in unfolded proteins in the ER. In yeast and mammals, eIF2α phosphorylation also initiates changes in gene expression promoted by proteins whose synthesis is paradoxically increased when there is increased eIF2α-P in cells, such as yeast GCN4 and ATF4 in mammals. Both are transcription factors that regulate the expression of hundreds of genes necessary for the cells to recover from the initial stress [12], [13], [14]. This type of selective transcriptional response is not present in trypanosomes [1].

Trypanosomes separated early from other eukaryotes and have some divergent features in the structure and regulation of eIF2. First, eIF2α has an amino terminal extension of >100 residues when compared with animals, fungi or higher plants. Second, the phosphorylated residue that aligns with S51 is threonine 169 (T169) in *Trypanosoma* sp. and threonine166 in *Leishmania* sp [15], [16], [17], [18]. This threonine residue is flanked by a sequence similar to that of S51 as part of a sequence motif required for recognition of eIF2α by eIF2α specific kinases [19]. *T. brucei* eIF2α T169 is phosphorylated *in vitro* by eIF2K2, one of the three eIF2α kinases in trypanosome genomes [15], [16], [17]. *In vivo*, this same residue is phosphorylated by eIF2K2 activated by different stress conditions in *T. cruzi* and *Leishmania*. In *Leishmania*, phosphorylation of eIF2α T166 has been shown to be necessary for the normal kinetics of differentiation of promastigotes into amastigotes but not for the process itself [15]. In addition, eIF2α T166 phosphorylation correlates with changes in the levels of translation that are observed during differentiation [20]. In *T. cruzi*, phosphorylation of eIF2α is also required for nutrient-induced differentiation from non-infective epimastigotes into infective metacyclic trypomastigotes, and again the phosphorylation of eIF2α T169 correlates with the levels of overall translation [18].

In *T. brucei* it has been shown that phosphorylation of eIF2α T169 is not necessary for either arrest or the formation of stress granules during heat shock [7]. Here, a *T. brucei* cell line containing a single eIF2α gene with a T169A mutation was used to determine whether phosphorylation of eIF2α T169 is necessary for either the growth arrest that occurs in stumpy bloodstream forms or the subsequent differentiation to procyclic forms. Both differentiation processes occurred with the same kinetics as the wild type control.

## 2 Materials and methods

### 2.1 Trypanosome strains and growth conditions

*T. brucei* EATRO 1125 bloodstream form cells were grown in HMI-9 medium salts [21] supplemented with 10% heat inactivated rabbit serum (Sigma) at 37 °C.

### 2.2 Western blots

1 × 10^7^ cells were harvested and washed twice in serum free HMI-9. The cells were resuspended in 100 μl SDS-PAGE sample buffer and incubated at 100 °C for 3 min. Standard SDS-PAGE and Western blotting procedures were used, rabbit anti-eIF2α was diluted 1:5000 [7]. For a loading control, anti-eIF2α antibodies were stripped off the membranes by incubation in 62.5 mM Tris-HCl, 100 mM β-mercaptoethanol, 2% SDS, (pH 6.7) at 50 °C for 30 min, and the membrane was re-probed with anti-tubulin antibodies [22].

### 2.3 Plasmids

The plasmid for deletion of the first allele of eIF2α and both plasmids for the substitution of the second allele for either the mutant or the wild type TbeIF2α have been previously described [7].

### 2.4 Transfection and selection of recombinant cells

Transfections were performed using 3 × 10^7^ cells resuspended in 100 μl of Amaxa Human T-cell buffer with 10 μg of linearized plasmid DNA in 0.2 mm cuvettes and program X-001 of the Amaxa Nucleofactor II (Lonza). Transfectants were selected and cloned in HMI-9 medium supplemented with G418 (2.5 μg/ml) and/or blasticidin (5 μg/ml) as required.

### 2.5 Screening of transfectants

After the antibiotic selection, clones were screened by amplification and sequencing of the region corresponding to the phosphorylation site region (T169). PCR was performed with Expand High Fidelity PCR (Roche) using oligonucleotides D526 (ATGGCAGCTTACGGTATAGTG) and D527 (AACCTCATTTCCCTTGAAGAA); the amplified region covered nucleotides 1–675 of the open reading frame. The PCR products were sequenced without cloning using oligonucleotide D527 as a primer.

### 2.6 *In vivo* differentiation from slender to stumpy bloodstream forms

CD1 mice were inoculated intraperitonially with between 5 and 15 × 10^5^ cultured bloodstream form trypanosomes. Four mice were used for each of the *eIF2A* T169A/- and *eIF2A* T169T/-cell lines. The subsequent parasitaemia was followed daily from day 2, trypanosome density measured using a haemocytometer after diluting 5 μl blood in 45 μl 0.83% ammonium chloride solution and further 10-fold dilutions as necessary.

### 2.7 *In vitro* differentiation from BSF to PCF

*In vitro* differentiation was initiated from blood collected 5 days after infection, as above. All populations were predominantly stumpy in morphology. The blood containing the parasites was incubated in DTM medium with 3 mM citrate/*cis*-aconitate at 28 °C at a density of 2 × 10^6^ trypanosomes/ml. After 6 h the red blood cells had settled and half the supernatant was removed to a new culture flask and proliferation rate was measured after 24 h.

### 2.8 *In vivo* differentiation from BSF to PCF

24 to 48 h post-eclosion and as the first feed, experimental tsetse flies (*Glossina pallidipes)* were fed with bloodstream form or cultured procyclics in washed red blood cells from horse (estimated 8 × 10^6^ parasites/ml), supplemented with 10 mM l-glutathione to increase infection rates [23]. Infected flies were maintained on sterile horse blood supplemented with 1 mM ATP and dissected 4–5 weeks post infection. Fly organs were dissected in separate drops of PBS and examined for the presence of trypanosomes in the salivary glands, proventriculus and midgut.

## 3 Results

### 3.1 Construction of a pleomorphic bloodstream form cell line expressing only eIF2α T169A

*T. brucei* EATRO 1125 bloodstream form cells were chosen for these experiments as they grow readily in culture and retain a pleomorphic phenotype in subsequent mouse infections. Trypanosomes are diploid and two sequentially modifications were used to make a cell line with a single copy of the eIF2α gene (*eIF2A*) that contained a T169A mutation as previously described [7]. First, the open reading frame in one allele was replaced with a blasticidin resistance open reading frame by homologous recombination to make *eIF2A*+/− cell lines. Second, a T169A mutation was introduced into the remaining allele, *eIF2A* T169A/-. This step altered the 3′ untranslated region of the *eIF2A* gene and a control cell line was made with the same change in the 3′ untranslated region but with wild type *eIF2A* open reading frame sequence, *eIF2A* T169T/-.

**Fig. 1:**
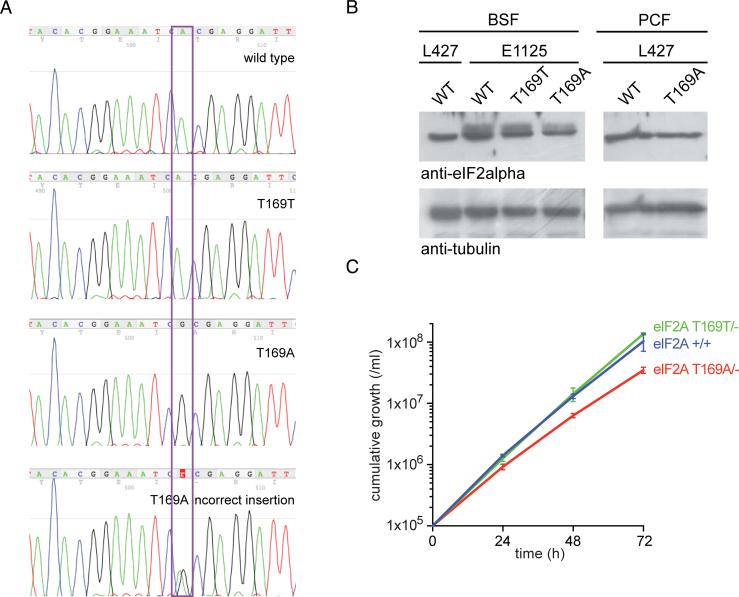
Characterization of cell lines by direct sequencing of the *eIF2A* locus, western blotting and growth rates. (A) Section of a sequencing trace of the PCR product amplified from genomic DNA corresponding to the part of the *eIF2A* gene encoding T169; the box indicates the mutated nucleotide. (B) Western blot of whole cell extracts of the wild type and mutant cell lines, tubulin is shown as a loading control. BSF, bloodstream forms; PCF, procyclic forms; L427, *T. brucei* Lister 427 cell lines; E1125, *T. brucei* EATRO1125 cell lines. (C) Cumulative growth curve of the indicated cell lines. *T. brucei* bloodstream form EATRO1125 wild type, control T169T and the mutant T169A. Bars indicate standard error (n = 3). Similar results were obtained in three different experiments.

After selection for expression of the drug resistant cassettes, clones were genotyped by PCR amplification of the *eIF2A* open reading frame from genomic DNA followed by direct sequencing of the PCR product (Fig. 1A). The mutation of the codon for T169, ACG to GCG, was readily distinguished and one clone that contained this mutation was recovered whereas several other clones produced amplicons that on sequencing contained a double peak for A and G and were discarded as being the product of alternative recombination events.

To test whether the expression levels of eIF2α were affected by the manipulations, a western blot of the selected clones using anti-eIF2α antibodies was performed [7] (Fig. 1B). Both the *eIF2A* T169T/- and *eIF2A* T169A/-cell lines had reduced expression when compared to the *eIF2A*+/+ control; this was expected as the gene copy number was reduced from two to one. There was no large difference in eIF2α expression levels between *eIF2A* T169T/- and *eIF2A* T169A/-cell lines. Two mobility forms of eIF2α were observed in all cell lines derived from the EATRO1125 isolate; the origin of the two forms is unclear as in another isolate, procyclic forms of Lister 427, a single mobility form was always observed (Fig. 1B).

### 3.2 Phenotype of eIF2A T169A/- cell line

To test whether the eIF2α T169A mutation affected the rate of proliferation, the growth of three cell lines was compared in culture: *eIF2A*+/+, *eIF2A* T169T/- and *eIF2A* T169A/-. The modifications to produce *eIF2A* T169T/- had no effect on growth rate when compared to *eIF2A*+/+ whereas the growth rate of the *eIF2A* T169A/- cell line was slightly slowed (Fig. 1C), with a population doubling time of 8.5 h compared to 7.2 h for *eIF2A*+/+ and *eIF2A* T169T/- cell lines. Thus, phosphorylation of eIF2α at T169 is not necessary for the growth of bloodstream form trypanosomes in culture. The same observation has been made previously for procyclic cells [7]. However, the availability of T169 for phosphorylation leads to increased proliferation in culture.

**Fig. 2:**
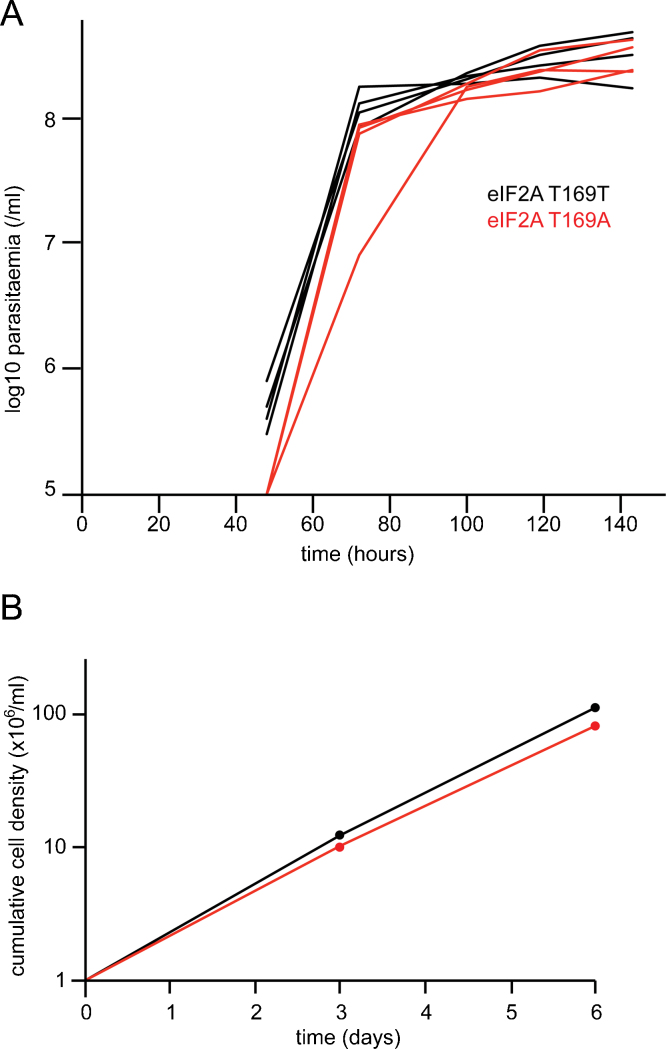
Phosphorylation of eIF2α T169 is not necessary for differentiation of slender to stumpy bloodstream forms or for differentiation to procyclic forms in culture. (A) Parasitaemia of individual mice measured at the indicated times after infection with *eIF2A* T169T (black n = 4) or *eIF2A* T169A (red n = 4) cell lines. (B) Cumulative growth curve of *eIF2A* T169T (black) or *eIF2A* T169A (red) procyclic cell lines obtained after *in vitro* differentiation of stumpy forms obtained from mice in (A). (For interpretation of the references to color in this figure legend, the reader is referred to the web version of this article.)

In animal infections, pleomorphic bloodstream form trypanosomes, such as the EATRO1125 isolate, use a quorum sensing mechanism based on the secretion of ‘stumpy induction factor' to restrict the maximum trypanosome cell density. In mice, the final density is affected by the genotype of both the trypanosome isolate and of the mouse. As the trypanosomes reach a threshold density a differentiation process is initiated that, over more than one cell cycle, results in a G1 cell cycle arrested form with a characteristic ‘stumpy' morphology and a plateau in the trypanosome density [24]. Some laboratory lines are unable to sense stumpy induction factor and the trypanosome density does not plateau but continues to increase until the mouse dies [24]. To test whether phosphorylation of T169 was necessary for this G1 arrest in response to stumpy induction factor, a set of mouse infections was performed with the two cell lines *eIF2A* T169T/- and *eIF2A* T169A/- and parasitaemia measured over a time course. Both cell lines showed an increase in parasitaemia until day 3 after which it was constant until day 5 when the experiment was ended (Fig. 2A). This kinetics of infection is characteristic of infections of mice with a trypanosome isolate that differentiates to stumpy forms [24]. On day 5, the vast majority of cells in both cells lines had a characteristic stumpy morphology. Thus, phosphorylation of eIF2α at T169 is not required for the growth arrest that occurs on differentiation of slender to stumpy bloodstream forms.

Stumpy bloodstream form cells differentiate to procyclic forms in response to environmental signals. These can be mimicked in culture by using differentiation medium supplemented with 3 mM sodium citrate and 3 mM sodium *cis*-aconitate and reducing the temperature to 28 °C [25]. On transfer to these conditions, cells remain arrested for 8–12 h whilst they undergo morphological transition and then start proliferating as procyclic forms. To test whether phosphorylation of eIF2α at residue T169 is necessary for this continued arrest during differentiation, blood was collected from mice that had been infected for 5 days with either *eIF2A* T169T/- or *eIF2A* T169A/- cell lines, as above, and the trypanosomes induced to differentiate. The outgrowth of procyclic cells was similar for the two cell lines and the subsequent growth of the two procyclic form cell lines in culture showed the same slight reduction in proliferation rate for the *eIF2A* T169A/- cells when compared to T169T/-cells (Fig. 2B). This small reduction in proliferation rate is not significant.

There are at least three further points in the life cycle that include a cell cycle arrest in G1. One occurs in procyclic cells as they migrate towards and invade the tsetse proventriculus [26]. Another occurs when the short epimastigote daughter cell remains in G1 until it reaches the salivary gland. A third occurs after differentiation in the salivary gland to the mammalian infective metacyclic forms, which remain arrested until introduced into a mammalian host. These steps have not yet been precisely reproduced in culture and so three cell lines, *eIF2A*+/+ (wild type), *eIF2A* T169T/- and *eIF2A* T169A/-, were used to infect tsetse flies to investigate these transitions in two different experiments (Table 1). In the first experiment, the flies were infected with bloodstream forms; the three cell lines showed similar progression with established infections in the midgut and proventriculus and trypanosomes of the expected morphologies (Table 1). No infections were detected in the salivary glands for any cell line in this experiment, including the wild type. To increase midgut infection rates and hence salivary gland infection rates, a second set of infections was initiated with procyclic forms of the three cell lines; in this case there was a significant difference in the number of salivary gland infections between the wild type and the two transgenic cell lines (Table 1). However, the small sample size for the two transgenic cell lines makes this difficult to interpret. This difference may reflect a decrease in the ability to progress through the developmental bottleneck at the proventriculus associated with the greater time in culture required for the production of transgenic cell lines. Alternatively, it is possible that the difference results from the lower expression levels of eIF2α but this is unlikely because both the transgenic cell lines *eIF2A* T169T/- and *eIF2A* T169A/- showed reduced ability to establish salivary gland infection. Taken together, these observations indicate that phosphorylation of eIF2α at T169 is not necessary for the differentiation as far as the proventriculus, including the cell cycle arrest prior to the asymmetric cell division.

## 4 Discussion

When a cell switches between quiescence and proliferation both the identity of proteins made and the rate of protein synthesis are altered. In trypanosomes, the non-selective polycistronic transcription of protein coding gene arrays means that there is an apparent conflict between maintaining/increasing the expression of proteins associated with quiescence and decreasing the overall rate of protein synthesis. Here, any role for phosphorylation of eIF2α on T169 in regulating differentiation between proliferative and quiescent developmental forms has been investigated. The main finding is that a cell line with a single *eIF2A* allele expressing a non-phosphorylatable eIF2α T169A was able to enter and exit quiescence in a similar manner to a wild type control. The ability of the mutant cells to enter and leave cell cycle arrest with wild type kinetics provides strong evidence that the phosphorylation of T169 is not sufficient to promote these transitions that are associated with changes in protein synthesis. The only phenotype observed was a slightly slower proliferation rate as bloodstream forms in culture.

In kinetoplastids, the evidence for a functional role for eIF2α T169 phosphorylation in regulating the overall rate of protein synthesis comes from *T. cruzi* and *Leishmania infantum*. In *T. cruzi* epimastigotes, the level of phosphorylation of T169 increases under a nutritional stress that results in differentiation to metacyclics, at the same time as the number of polysomes decreases, providing evidence that the overall rate of protein synthesis could be regulated by eIF2α phosphorylation. Overexpression of eIF2α mutated in the phosphorylation site has a dominant effect and reduced differentiation from the proliferative epimastigote to the non-proliferative metacyclic form [18]. In *Leishmania*, a slowing in growth associated with differentiation of promastigotes to amastigote forms also coincides with eIF2α phosphorylation and overexpression of an eIF2α kinase deleted for its regulatory domain and sequences necessary for correct localization slowed differentiation [15]. These observations indicate that phosphorylation of eIF2α is almost certainly necessary for the starvation response in *T. cruzi* and are consistent with a role for eIF2α phosphorylation in the differentiation of *Leishmania* after the uptake by macrophages and acidification in the parasitophorous vacuole.

In contrast, the differentiation of proliferating *T. brucei* slender to non-proliferating stumpy forms does not occur as a consequence of nutritional starvation or environmental changes. Similarly, the formation of procyclics in the insect gut does not appear to depend on the lack of nutrients [4]. Our results are then consistent with these life cycle differences between *T. brucei* and the other studied kinetoplastids, and they represent the first direct evidence indicating that phosphorylation of eIF2α T169 is not necessary for controlling these *T. brucei* differentiation events [27]. Our studies further suggest that the drastic decrease in polysomes observed in stumpy forms is not due to the phosphorylation of eIF2α T169. However, we cannot rule out that the kinetoplastid eIF2α N-terminal extension, absent in other eukaryotes, has other phosphorylation sites able to regulate translation initiation which could compensate for the absence of T169. It is also possible that the decrease in protein synthesis initiation in these forms may involve the inhibition of the function of the eIF4F complex. Trypanosomatids express several orthologues of eIF4E and eIF4G, some of them shown to be involved directly in initiation of translation [28].

Given the relevance of phosphorylation of eIF2α at T169, or equivalent residue, in differentiation events in other parasites, it is possible that this phosphorylation and consequent inhibition of translation could be required for the *T. brucei* transformation of epimastigotes to metacyclics in the insect salivary gland, a differentiation process that occurs in an environment that is poor in nutrients.

## Figures and Tables

**Table 1:** Progression of infections with eIF2 mutants in the tsetse fly.^a^

Stage infected	eIF2α Genotype	Days post infection when dissected	Number of flies dissected	Number with midgut infection	Number with midgut infection that also had an infection in:	
					Proventriculus	Salivary glands
BSF	Wild type	28–35	40	27/40	22/26	0/27
	T169T	28–35	34	15/34	10/10	0/15
	T169A	28–35	74	35/74	21/33	0/35
PCF	Wild type	30–33	35	28/35	28/28	11/28
	T169T	30–33	14	12/14	10/10	1/12
	T169A	30–33	14	10/14	7/8	0/10

a Flies were infected with wild type, eIF2A T169T or eIF2A T169A cell lines, either as bloodstream or as procyclic forms. After the indicated number of days, midgut, salivary glands and proventriculus were dissected, and the number of infected tissues determined.
